# “What’s the evidence?”—Towards more empirical evaluations of the impact of OR interventions in healthcare

**DOI:** 10.1080/20476965.2020.1857663

**Published:** 2020-12-15

**Authors:** Guillaume Lamé, Sonya Crowe, Matthew Barclay

**Affiliations:** a The Healthcare Improvement Studies Institute (THIS Institute), University of Cambridge, Cambridge, UK; b Laboratoire Génie Industriel, Université Paris-Saclay, CentraleSupélec, Gif-sur-Yvette, France; c Clinical Operational Research Unit, University College London, London, UK

**Keywords:** Health Quality and Evaluation, healthcare Improvement Research, impact

## Abstract

Despite an increasing number of papers reporting applications of operational research (OR) to problems in healthcare, there remains little empirical evidence of OR improving healthcare delivery in practice. Without such evidence it is harder both to justify the usefulness of OR to a healthcare audience and to learn and continuously improve our approaches. To progress, we need to build the evidence-base on whether and how OR improves healthcare delivery through careful empirical evaluation. This position paper reviews evaluation standards in healthcare improvement research and dispels some common myths about evaluation. It highlights the current lack of robust evaluation of healthcare OR and makes the case for addressing this. It then proposes possible ways for building better empirical evaluations of OR interventions in healthcare.

## 1. Introduction

Many operational research (OR) practitioners and academics involved in healthcare will have been confronted with the comment “I like the idea of OR, but what’s the evidence for it?” when talking to clinicians, funders, policy-makers or editors and reviewers in medical journals. With this question, they are asking for evidence of OR improving healthcare organisations and often expect this evidence to have a certain form and be generated through specific evaluation processes.

Research applying OR techniques (including Soft OR and Problem Structuring Methods) to improve healthcare delivery is developing rapidly (e.g., Brailsford et al., 2009; Zhang et al., 2018), but falls short in demonstrating impact through sound empirical evaluations. Most healthcare OR papers do not discuss implementation (Brailsford et al., 2009; Brailsford & Vissers, 2011), let alone assess the *impact* of the OR intervention. Therefore, we have little to support the potential of OR in bringing about desirable change to the quality, safety, and efficiency of healthcare delivery, and lack key information to learn and iteratively refine our approaches.

Evaluation helps decision-makers understand what works in a given context, as well as how and why it works, before choosing a course of action (The Health Foundation, 2015). In this viewpoint, we argue that we need more, and better, evaluations of the impact of OR interventions in healthcare. We take a broad view of OR, including but not limited to Problem Structuring Methods, mathematical modelling and simulation (see (Pitt et al., 2016) for examples). First, we discuss the type of evidence currently generated to support claims of OR’s potential impact. We then highlight the gap between the objectives of evidence-based healthcare improvement and the reality of current practice in healthcare OR. Finally, we identify possible ways to address this gap.

## 2. Evaluating healthcare improvement interventions

Evaluations aim “to determine merit, worth, value or significance” (Patton et al., 2014). In the context of interventions for improving healthcare, evaluation means answering the questions (Walshe, 2009):

- *Does the intervention work, and how well? How much does it cost?* The efficiency, effectiveness and efficacy of the intervention needs to be assessed to know if the resources invested are well spent. This is referred to as *outcome evaluation*.
- *Why and how does the intervention generate these outcomes in this context?* Underlying mechanisms linking an intervention to a given set of outcomes need to be analysed to understand in which other circumstances the intervention (or parts of it) may be useful. This is labelled *process evaluation*.
- *What is it like to use the intervention?* Learning from practical implementation by exploring the experience of those implementing and participating in the intervention, to improve the implementability and feasibility of the intervention.

The emerging consensus among healthcare improvement evaluators is to use a “programme theory” to plan the evaluation and synthesise its results. Programme theories synthesise different types of knowledge to explain how the components of an intervention will generate a certain effect through intermediate processes and subject to moderating factors (Davidoff et al., 2015; Funnell & Rogers, 2011).

The key advantage of a theory-driven approach is that it goes beyond asking whether the intervention works, to exploring where it works, for whom, and why, through unpacking the mechanisms that generate these impacts. This makes it easier to understand to what extent success is transferrable, not least by assessing what aspects of the intervention are particularly contingent on context (Davidoff et al., 2015). Programme theories also help explain unsuccessful interventions (Funnell & Rogers, 2011).

Programme theories can be used in quantitative, qualitative or mixed-methods evaluations, and in experimental, quasi-experimental or observational designs. In general, a degree of pragmatism is considered necessary in choosing an appropriate evaluation design due to the complexity of some interventions, their dynamic and evolving character, and the cost of running multisite experimental studies (Barry et al., 2018). Nonetheless, this flexibility does not mean that all evaluation designs and methods are equivalent or provide the same level of confidence in the impact of the intervention. For example, simple uncontrolled before-and-after studies are a very weak form of evaluation since any changes observed may be caused by secular trends, changes in the environment or the phenomenon of “regression to the mean”, rather than the intervention (Eccles et al., 2003). Interrupted time-series designs, where the outcomes of interest are measured at several points before, during, and after the intervention, are a stronger design because they allow the effect of the intervention to be distinguished from secular trends, while remaining simpler to organise than controlled experiments (Fretheim & Tomic, 2015). Although the literature tends to discuss quantitative designs more extensively, qualitative studies are also part of an evaluator’s toolkit in healthcare improvement (Portela et al., 2015).

In the Appendix to this article, we dispel some common myths about the evaluation of healthcare improvement interventions along with illustrative examples.

## 3. The current state of evaluation in healthcare OR

Much of the healthcare improvement research literature focuses on publishing evaluations but the healthcare OR literature has largely evolved separately. Evaluations of OR interventions are rarely reported. OR case studies typically describe the process of building models and improving their performance with few papers mentioning implementation. Literature reviews have highlighted this in healthcare OR in general (Brailsford & Vissers, 2011; Mahdavi et al., 2013; Van Sambeek et al., 2010), in modelling and simulation studies (Brailsford et al., 2009; Fone et al., 2003; Jahangirian et al., 2012; Long & Meadows, 2018; Mohiuddin et al., 2017; Van Lent et al., 2012; Wilson, 1981), multi-criteria decision analysis (Marsh et al., 2014), the application of Soft Systems Methodology (Augustsson et al., 2019), optimisation (Ahmadi-Javid et al., 2017), scheduling (Marynissen & Demeulemeester, 2019; Samudra et al., 2016) or in specific areas of healthcare, such as outpatient chemotherapy (Lamé et al., 2016), global health (Bradley et al., 2017) or community healthcare (Palmer et al., 2018). The results of Brailsford et al. (2009) that only 5 to 8% of modelling and simulation papers in healthcare mention the implementation of results in practice seem to hold for healthcare OR more broadly.

OR researchers and practitioners sometimes argue that the learning generated through an OR project is more important than the “answers”, with stakeholders gaining understanding through the process about how their organisations work and what affects their performance (Sterman, 1994). However, we still need to assess who learns what, and how (Lamé & Simmons, 2020). Some researchers have started to do so through lab experiments (Monks et al., 2014), or retrospective interviews with experts (Thompson et al., 2016). Empirical evaluations in real improvement projects have also been published in a corporate context (Cavaleri & Sterman, 1997; Read et al., 2012), but remain rare. In many cases, there is no measurement of changes to participants’ behaviour that could be linked to modelling projects, nor of participants’ reactions to and opinions of the modelling effort (Kunc et al., 2018). There also does not appear to be a consensus on what constitutes learning in OR interventions or on how to assess it.

## 4. The gap between standards of evaluation in healthcare improvement and current practice in healthcare OR

The lack of robust evaluation of the impact of OR interventions is particularly problematic in healthcare, where the evidence-based paradigm is spreading from clinical practice to management and policy, leading to increasing pressure for better evidence on what works to improve healthcare delivery (Auerbach et al., 2007; Bevan et al., 2005; Grady et al., 2018; The Health Foundation, 2015). In Section 2, we described the standards for evaluation of healthcare improvement interventions. The gap between these standards and current practice in healthcare OR raises pragmatic and scientific arguments for empirical evaluations of the impact of OR interventions in healthcare organisations:

- Research on OR interventions in healthcare must produce evidence that is acceptable to those who will use the results, providing empirical evidence to meet the needs and expectations of healthcare improvement funders, practitioners and policy-makers.
- Expectations for sound evidence that OR brings about improvement are healthy and should drive us to better evaluate interventions not only as an end in itself, but as a means to enhance the effectiveness of the work we do in bringing about positive change in healthcare.

Empirical data on observed changes (or absence of such changes) linked to OR interventions is needed so that we can understand their impact. Recent examples show this is not out-of-reach. For instance, Monks et al. (2015) present a case study of the impact of a simulation study in emergency stroke care combining different types of quantitative evidence (before-and-after analysis on different process duration metrics, time series analysis on the implementation of certain good practices) with an analysis of the modeller’s field notes during the intervention. Outcomes of interest improved after the intervention and the time-series analysis gives confidence that this was attributable to the intervention, whilst the qualitative evidence helped identify aspects of the intervention that supported stakeholder engagement and the credibility of the results. In another example, Crowe et al. (2017) use ethnography to provide insights into the role and specific contribution of OR in multidisciplinary projects in healthcare. Other studies have also started to look at the cost-effectiveness of building a model to tackle issues in healthcare (Soorapanth & Young, 2019; Young et al., 2018). Impact evaluations of Soft OR approaches are also appearing (Emes et al., 2018).

**Figure 1.**
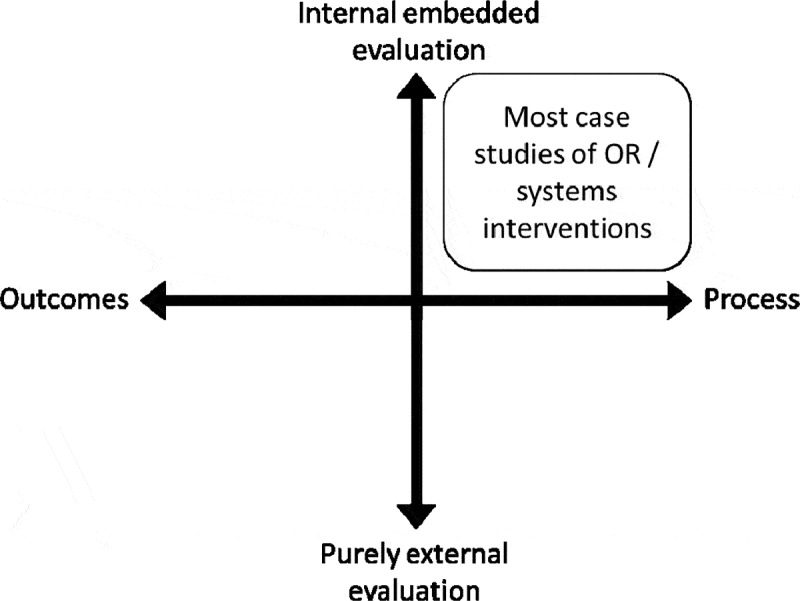
Positioning current evaluations of OR approaches in a landscape of evaluation approaches.

Another salient aspect of current healthcare OR publications is that the evaluator is often *embedded* in the intervention team (Barry et al., 2018). Many papers are self-reports of interventions carried out by the authors, with very few *external* evaluations of OR projects. The stroke project mentioned above (Heaton et al., 2016) is an exception, albeit aimed at evaluating the funding initiative that supported the project rather than the OR intervention. In another case, the design of the UK NHS Direct national 24-hour telephone helpline service, an external evaluation was reported independently from the OR intervention itself (Munro et al., 2000). However, the external evaluation focused on the solution proposed by the OR project (the telephone helpline) and does not mention the OR intervention. Therefore, it is not possible to understand from this evaluation how the OR intervention affected decisions that led to the implementation of the chosen solution. Elements on the role played by operational researchers in this project have been reported by the OR team (Royston et al., 2003), but fall into the category of self-reports rather than external evaluations. Existing systematic reviews of the literature, detailed in Section 3, show most evaluations of healthcare OR are reported by the operational researchers who led the intervention, and focus on the modelling stages rather than the implementation of the findings. Current OR evaluations published in the academic literature primarily cover only one of the four quadrants of possible evaluation modalities (Figure 1):

- We rarely look at the *outcomes* of our interventions,
- We rarely design *external* evaluations.

There is nothing wrong with internal process evaluations of OR interventions, which can address many interesting questions. The problem comes when we avoid other types of evaluation. By not measuring what happens after our interventions, or relying on self-reported data and internal evaluations, we risk introducing biases that make interventions appear useful when they might do little to solve issues in practice. In reality, many interventions do not work as well as planned or equally well in every context (Øvretveit, 2011), yet few unsuccessful studies are reported in OR journals (for examples of OR interventions in healthcare reported as partially unsuccessful by their authors, see Bennett & Worthington, 1998; Connell, 2001).This suggests either some form of publication bias (where only positive studies are reported), or outcome reporting bias (where reporting is biased towards the more positive aspects of interventions, overlooking less successful dimensions) (Fanelli, 2012).

External evaluation and outcome evaluations are not panaceas. They are not always needed by healthcare stakeholders, nor are they always appropriate. Yet including more of them in our research portfolio could help us to understand how OR interventions work, specify which work best in different settings, and anticipate their likely impact. Systematic evaluation and reporting would also allow us to critique and improve the interventions we develop and our practice as operational researchers. Ultimately, these types of evaluation would provide stronger arguments for using OR methods to bring about improvement in healthcare organisations, and enable continuous learning and improvement.

## 5. Towards empirical evaluations of OR in healthcare

### 5.1. What do we need to evaluate?

How should we explore the other quadrants of Figure 1 and what exactly do we need to evaluate in OR interventions? When using OR methods to structure, model and better understand a problem situation in order to take informed action, the intervention and potential outcomes are harder to define upfront than for, say, an annual training module on patient safety. For instance, an OR intervention may use simulation to understand patient flows in an emergency department and evaluate the impact of different ways of organising resources and processes. The proposed changes from this OR intervention (e.g., shifting resources across different parts of the pathway) will then be debated and either implemented or not, and if implemented may or may not bring improvement.

Determining whether improvement has occurred is not always straightforward and notions of improvement may be contested by different stakeholders. Therefore specifying appropriate evaluation outcomes for OR interventions can be challenging, particularly when the situations being tackled are multi-faceted and messy (Williams, 2008). However, some OR interventions in healthcare aim to address relatively bounded issues, such as patient flow (Mohiuddin et al., 2017; Palmer et al., 2018), for which indicators can be defined and used to measure impact. In more complex situations, qualitative methods may be better suited to assessing whether and in which ways things improve or worsen.

**Figure 2.**
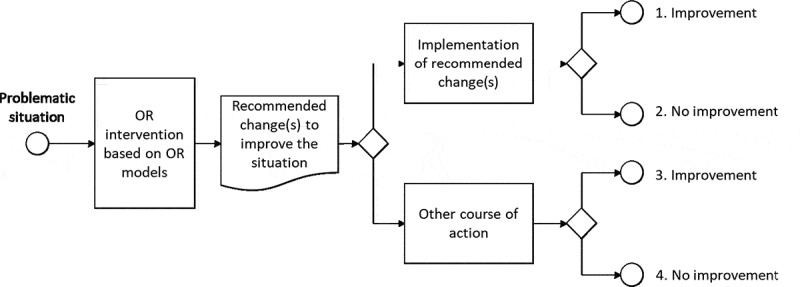
Generic model of the outcomes of OR interventions (similarities can be noted with the four stages of success in simulation projects suggested by Robinson and Pidd, 1998).

A simple model of the possible outcomes from an OR intervention can be helpful when planning evaluations. For example, Figure 2 shows a generic model appropriate for relatively simple, linear, OR interventions. It raises generic questions for each possible outcome:

1. The changes recommended by the OR intervention are implemented, and the situation improves. Can the improvement be attributed to the implementation of the recommended changes? Can the choice to implement these changes be attributed to the OR intervention (or would it have been chosen anyway)? Did the recommended changes generate side effects?

2. The changes recommended by the OR intervention are implemented, but the situation does not improve. Was the decision to implement these changes attributable to the OR intervention? Were the recommended changes implemented correctly? Was the OR model appropriate (complexity, type of model, hypotheses, factors included and excluded)? Did the recommended changes generate side effects?

3. The changes recommended by the OR intervention are not implemented, and the situation improves. Why were the recommended changes not chosen? Might the recommended changes have led to greater improvement?

4. The changes recommended by the OR intervention are not implemented, and the situation does not improve. Why were the recommended changes not chosen?

Addressing these questions requires a range of evaluation methods. For example, matters of attribution (“did the improvement come from the intervention?”) can be approached using experimental or quasi-experimental quantitative methods (e.g., using Statistical Process Contro over the course of the modelling process (Perla et al., 2011)), while qualitative methods (e.g., interviews and observations) are well suited to identifying why people did or did not accept the OR recommendations. Side effects can be investigated both qualitatively and quantitatively, either by assessing against pre-identified potential side effects or in a more exploratory manner.

Existing frameworks for evaluating “complex interventions” in healthcare (like that proposed by the UK Medical Research Council, c.f. Campbell et al., 2007) may provide a useful basis on which to develop evaluation frameworks for OR interventions. Useful insights might also be drawn from the growing field of Behavioural OR, which seeks to examine the role and impact of behaviour on the use of OR to support decision-making (Franco & Hämäläinen, 2016), including through qualitative approaches and experimental designs that could help us to understand stakeholders’ acceptance of, and fidelity to, recommendations from OR interventions.

### 5.2. Programme theories for OR interventions

A programme theory offers a theoretical model of how an intervention is expected to generate certain outcomes in a given context. Programme theories can help in designing appropriate evaluations. Figure 2 stems from a simple generic programme theory for OR, with three elements:

- Modelling supports the establishment and sharing of a common, simplified, representation of a complex situation.
- Manipulation of this simplified representation allows assessment of the likely effect of changes, and an exploration of the importance of different factors in the overall behaviour of the system.
- This experimentation allows people to learn about the behaviour and dynamics of a system and gives a shared foundation for debate, leading to better decisions about how to make changes in their organisation.

This simple programme theory will not always be appropriate. Many OR projects are iterative or involve continuous negotiation (Williams, 2008), so the programme theory would need to reflect this. There may need to be additional steps addressing the construction and presentation of the model: for example, some would argue that building models collaboratively, in a facilitated environment, improves their acceptance by stakeholders and that effective visualisation of model outputs can affect how users perceive the model. Fine-tuning an evaluation requires all these elements be considered and integrated into a programme theory that reflects the specifics of the OR intervention and the context.

We will often be able to develop programme theories based on our understanding of OR methods and the context in which they will be applied, but theoretical approaches from other disciplines also offer a rich repertoire for building programme theories for OR interventions. Activity theory (Leroy White et al., 2016), the concept of boundary objects (Franco, 2013), the mangle of practice framework (Ormerod, 2014) and single and double-loop learning (Monks et al., 2014) have all been used for this purpose, albeit not in healthcare OR. Drawing on theory from the social sciences forms a key aspect of the Behavioural OR research agenda (Becker, 2016; Brocklesby, 2016).

### 5.3. Practical challenges to evaluating OR

Clarifying the scientific challenge and providing elements of a programme theory are merely first steps towards more empirical evaluations of OR interventions in healthcare, and challenges in practical attempts to evaluate healthcare OR may well occur.

For example, evaluation of OR may not fall within the remit of many traditional **funding** sources for OR projects. However, evaluations have started to appear in the UK, funded by organisations such as the National Institute for Health Research’s Applied Research Collaborations (NIHR ARCs, previously CLAHRCs) and the Health Foundation (e.g., Crowe et al., 2017; Monks et al., 2015).

Most OR researchers are not trained in the **standards, methods and practices** of evaluating healthcare improvement interventions. In this case, collaborating with evaluation-driven disciplines such as health services research, implementation science or improvement research can help (Brailsford & Klein, 2015), and there may be scope to incorporate evaluation techniques within OR degrees and professional development. This resonates with one stream of the Behavioural OR movement, seeking to use theories and methods from the social and behavioural sciences to better understand what happens in and around OR interventions (Becker, 2016; Brocklesby, 2016).

Even so, **methodological challenges** arise when evaluating complex, evolving interventions (Burke & Shojania, 2018). Specific frameworks may need to be developed for OR interventions, depending on the type of problem, the modelling methods used and the scope of the project.

Finally, robust evaluation of OR methods and interventions may need multisite projects to compare the effect of an intervention in different places and contexts, requiring different **research project management skills and strategies** from the one-off projects that constitute the majority of applied OR papers at present.

Although there is no “one best way” that would apply to every OR method and every situation in healthcare, some practical recommendations can be identified (Box 1).

**BOX 1.** Recommendations

Think about *interventions* rather than models or methods. The impact of OR interventions can be very diverse, with models being only one aspect (Crowe et al., 2017). The processes of interventions, the interactions with the team and the social and political dynamics at play should also be assessed. Think about evaluation from the beginning.Develop a monitoring and evaluation plan when designing OR interventions. What would success look like for this intervention? How could it be assessed? What could go wrong? What type of data is needed for the evaluation?Include follow-up periods in projects to assess the impact of OR interventions. Collaborate with specialists in qualitative and quantitative evaluation to frame and conduct evaluations. Effective models exist for such collaborations between intervention designers, implementers and evaluators (Brewster et al., 2015). Use a programme theory to model interventions and design their evaluation. Theory-driven evaluation is in line with past recommendations in OR (Midgley, 1998; White, 2006) and the Behavioural OR movement (Becker, 2016; Brocklesby, 2016) has explored the type of external theories that can be drawn upon. Adapt evaluation designs to interventions, contexts of implementation and resources (Eccles et al., 2003; Portela et al., 2015). Think in research programmes rather than projects, with replication of the same intervention across multiple sites. This allows comparisons of the effect of the same intervention (e.g., a group model-building approach, an optimisation strategy or a problem structuring intervention) across settings. Incorporate evaluation in OR training curricula, including creating professional development modules on evaluating OR. Support the publication of empirical evaluations in OR journals and prompt authors to mention the practical outcome of OR interventions in their articles or to write follow-up articles on the implementation (or lack thereof).

## 6. Conclusion

Promoting the value of healthcare OR is challenging, despite steady developments since the 1950’s and areas of notable success (for example, embedded OR in the UK NHS, c.f. Royston et al., 1999, 2003), because we rarely evaluate the impact of our projects. A key limitation in this analysis is our reliance on the peer-reviewed academic literature. Indeed, there could be OR practitioners generating very good evidence of the impact their OR interventions have on the organisations they are working with, enabling them to illustrate the value of the techniques within their organisation or to their next potential client. However, we are not aware of such evidence in the grey literature, and, if kept confidential, such evidence is of little use to the wider OR community.

Failing to engage in evaluation limits our impact on practices and performance in healthcare, not least because we are less able to have meaningful conversations with healthcare professionals who increasingly seek an evidence-base for change. Systematic approaches to assess the impact of our efforts are required, and there are already a range of approaches for evaluating healthcare improvement interventions that could be adapted for use with OR interventions. Evidence is not everything, and having it will not suddenly change how OR is perceived and used in healthcare, but not having it makes OR easy to dismiss. Importantly, beyond promoting OR methods, evidence from evaluations would allow us to learn about their use in practice so that we can improve their effectiveness.

In this article, we have reviewed recommended practice in healthcare improvement research, which combines approaches prevalent in health services research, social sciences and public policy. We do not mean to place these approaches on a pedestal, nor suggest that healthcare improvement research is only of the highest methodological quality (Auerbach et al., 2007). Rather, we wish to trigger a debate on what constitutes evidence that OR is effective in bringing about desirable changes in healthcare. Many questions remain open. What outcomes should we measure? What are the key mechanisms that make OR interventions effective? We often talk about learning as a key process in OR projects, but how can we operationalise this concept for evaluation? On a more mundane level, how can this enterprise be funded? Who should we partner with? All these questions open exciting avenues for experimentation and progress for the healthcare OR community.

## Supplementary Material

Supplemental Material
