# Supplementary material for: “What’s the evidence?”—Towards more empirical evaluations of the impact of OR interventions in healthcare
Source: Health Syst (Basingstoke). 2020 Dec 15;11(1):59–67. doi: 10.1080/20476965.2020.1857663 (PMC8812794; doi:10.1080/20476965.2020.1857663)
Supplement: Supplemental Material [file THSS_A_1857663_SM6760.docx]

# Appendix A: Four common misconceptions about the evaluation of interventions to improve healthcare delivery

**1. When someone says “evaluation” in healthcare, it always means, “randomised controlled trial” (RCT).**

Experimental methods, and RCTs in particular, remain perceived by some as the gold standard for evaluating medical interventions (Grady, Redberg, & O’Malley, 2018). However, controlled trials can be impractical to implement and sometimes yield disappointing results, for instance when they are undertaken too soon on interventions that are not fully understood (Shojania, 2013). For these reasons, evaluation scientists have long since accepted other methods (Patton, 2018). For example, quasi-experimental and observational approaches such as Statistical Process Control or Interrupted Time Series have proven to be useful in assessing the effectiveness of interventions (Fretheim & Tomic, 2015; Wong & Shojania, 2018). Qualitative methods also have a role to play in evaluating improvement interventions (Pope, van Royen, & Baker, 2002), such as clarifying the mechanisms that make interventions successful or analysing how implementation is affected by contextual factors. In this multimethod approach, RCTs may become appropriate at the end of a process of intervention refinement and theory-generation (Shojania, 2013).

**2. Evaluation can only assess the effectiveness of simple, bounded interventions, like medications.**

Simpler (well-identified, easily replicable and standardisable) interventions are naturally easier to evaluate. However, the evaluation of complex interventions has received specific methodological attention (Campbell et al., 2007; Rogers, 2008). For example, a project that combined quantitative and qualitative methods to evaluate an audit-and-feedback intervention in maternity care showed a positive impact on some outcomes, and allowed the identification of drivers and barriers mediating the improvements (Reszel et al., 2019).

**3. Evaluation studies only look at clinical patient outcomes and overlook organisational impacts and the processes of implementing interventions.**

Ultimately, improvement efforts in healthcare aim to improve outcomes for patients, either directly or through saving resources that can be invested elsewhere. However, since most interventions will only indirectly affect patient outcomes such as survival rates, many evaluations assess organisational outcomes (Brown et al., 2008). Many studies have also looked at how interventions are received and implemented. This is sometimes called “process evaluation” (Hulscher, Laurant, & Grol, 2003). It can be embedded in trials (Oakley, 2006), or be conducted independently. Understanding how local contexts affect intervention delivery is crucial in these studies (Øvretveit, 2011). This is often done through qualitative methods (Portela, Pronovost, Woodcock, Carter, & Dixon-Woods, 2015), which can then help in understanding to which contexts the evaluation results can be generalised.

**4. Evaluation-driven studies invariably embrace a positivist stance.**

Evaluation science embraces a variety of paradigms (Patton, 2018). In healthcare improvement research, evaluations informed by critical realism (Burton, Rycroft Malone, Robert, Willson, & Hopkins, 2014) and complexity-informed perspectives (Braithwaite, 2018) have gained traction recently, including to study OR-type interventions like computer simulation (Long, McDermott, & Meadows, 2019). Nonetheless, it is true that these paradigms remain less prominent, and that many practitioners and academics in health services still refer to so-called “hierarchies of evidence”, with experimental approaches inspired by clinical evaluation, and most importantly RCTs, sitting at the top (Murad, Asi, Alsawas, & Alahdab, 2016).

.

## References

Braithwaite, J. (2018). Changing how we think about healthcare improvement. *BMJ, 361*, k2014. doi:10.1136/bmj.k2014

Brown, C., Hofer, T., Johal, A., Thomson, R., Nicholl, J., Franklin, B. D., & Lilford, R. J. (2008). An epistemology of patient safety research: a framework for study design and interpretation. Part 3. End points and measurement. *Quality and Safety in Health Care, 17*(3), 170-177. doi:10.1136/qshc.2007.023655

Burton, C. R., Rycroft Malone, J., Robert, G., Willson, A., & Hopkins, A. (2014). Investigating the organisational impacts of quality improvement: a protocol for a realist evaluation of improvement approaches drawing on the Resource Based View of the Firm. *BMJ Open, 4*(7), e005650. doi:10.1136/bmjopen-2014-005650

Campbell, N. C., Murray, E., Darbyshire, J., Emery, J., Farmer, A., Griffiths, F., . . . Kinmonth, A. L. (2007). Designing and evaluating complex interventions to improve health care. *BMJ, 334*(7591), 455-459. doi:10.1136/bmj.39108.379965.BE

Fretheim, A., & Tomic, O. (2015). Statistical process control and interrupted time series: a golden opportunity for impact evaluation in quality improvement. *BMJ Quality & Safety, 24*(12), 748-752. doi:10.1136/bmjqs-2014-003756

Grady, D., Redberg, R. F., & O’Malley, P. G. (2018). Quality improvement for quality improvement studies. *JAMA Internal Medicine, 178*(2), 187-187. doi:10.1001/jamainternmed.2017.6875

Hulscher, M. E. J. L., Laurant, M. G. H., & Grol, R. P. T. (2003). Process evaluation on quality improvement interventions. *Quality and Safety in Health Care, 12*(1), 40-46. doi:10.1136/qhc.12.1.40

Long, K. M., McDermott, F., & Meadows, G. N. (2019). Factors affecting the implementation of simulation modelling in healthcare: A longitudinal case study evaluation. *Journal of the Operational Research Society*, 1-13. doi:10.1080/01605682.2019.1650624

Murad, M. H., Asi, N., Alsawas, M., & Alahdab, F. (2016). New evidence pyramid. *Evidence-Based Medicine, 21*(4), 125-127. doi:10.1136/ebmed-2016-110401

Oakley, A. (2006). Process evaluation in randomised controlled trials of complex interventions. *BMJ, 332*(7538), 413-416. doi:10.1136/bmj.332.7538.413

Øvretveit, J. (2011). Understanding the conditions for improvement: research to discover which context influences affect improvement success. *BMJ Quality & Safety, 20*(Suppl 1), i18-i23. doi:10.1136/bmjqs.2010.045955

Patton, M. Q. (2018). Evaluation Science. *American Journal of Evaluation, 39*(2), 183-200. doi:10.1177/1098214018763121

Pope, C., van Royen, P., & Baker, R. (2002). Qualitative methods in research on healthcare quality. *Quality and Safety in Health Care, 11*(2), 148-152. doi:10.1136/qhc.11.2.148

Portela, M. C., Pronovost, P. J., Woodcock, T., Carter, P., & Dixon-Woods, M. (2015). How to study improvement interventions: a brief overview of possible study types. *BMJ Quality & Safety, 24*(5), 325-336. doi:10.1136/bmjqs-2014-003620

Reszel, J., Dunn, S. I., Sprague, A. E., Graham, I. D., Grimshaw, J. M., Peterson, W. E., . . . Walker, M. C. (2019). Use of a maternal newborn audit and feedback system in Ontario: a collective case study. *BMJ Quality & Safety*, bmjqs-2018-008354. doi:10.1136/bmjqs-2018-008354

Rogers, P. J. (2008). Using Programme Theory to Evaluate Complicated and Complex Aspects of Interventions. *Evaluation, 14*(1), 29-48. doi:10.1177/1356389007084674

Shojania, K. G. (2013). Conventional evaluations of improvement interventions: more trials or just more tribulations? *BMJ Quality & Safety, 22*(11), 881-884. doi:10.1136/bmjqs-2013-002377

Wong, B. M., & Shojania, K. G. (2018). Rigor in quality improvement studies and the role of time-series methodologies. *JAMA Internal Medicine, 178*(5), 724-725. doi:10.1001/jamainternmed.2018.0863
